# Physical activity and non-suicidal self-injury in Chinese adolescents: a two-wave study of anxiety symptoms and social support

**DOI:** 10.3389/fpubh.2026.1980538

**Published:** 2026-10-29

**Authors:** Xinyue Pan, Jianyi Li, Ting Zhou, Qi Zhou

**Affiliations:** 1 School of Physical Education, China University of Mining and Technology, Xuzhou, Jiangsu, China; 2 Medical Technology Department, Guizhou Nursing Vocational College, Guiyang, Guizho, China; 3 School of Physical Education and Health, Wenzhou University, Wenzhou, Zhejiang, China

**Keywords:** adolescents, anxiety, longitudinal study, non-suicidal self-injury, physical activity, social support

## Abstract

**Background:**

Non-suicidal self-injury (NSSI) is common during adolescence and is associated with substantial psychological burden and elevated subsequent suicide risk. Physical activity (PA) may be a modifiable correlate of NSSI, but prospective evidence on potential emotional mechanisms and social boundary conditions remains limited. This study examined whether baseline PA was associated with subsequent NSSI, whether anxiety symptoms accounted for part of that association, and whether baseline social support moderated the relevant pathways.

**Methods:**

A school-based two-wave cohort of 756 Chinese adolescents and young students (mean age = 17.36 years, SD = 2.58; 51.7% female) was followed for 6 months. At Time 1 (T1), participants reported PA, social support, anxiety symptoms, NSSI, and demographic characteristics; anxiety and NSSI were reassessed at Time 2 (T2). Autoregressive regression models adjusted for baseline anxiety or NSSI as appropriate, together with age, gender, and urban/rural residence. Indirect effects were estimated with 5,000 bootstrap resamples, and interaction terms were used to examine first- and second-stage moderation.

**Results:**

Higher T1 PA was associated with lower T2 NSSI after adjustment for T1 NSSI. The indirect association through T2 anxiety was −0.12 [95% CI (−0.18, −0.06)], representing 48.0% of the reported total association. T1 social support significantly moderated the T1 PA-T2 anxiety association (*p* = 0.003); simple-slope estimates indicated a stronger inverse PA-anxiety association at higher social support. Social support did not significantly moderate the T2 anxiety-T2 NSSI association (*p* = 0.281).

**Conclusion:**

The findings are consistent with a model in which greater PA is prospectively associated with lower NSSI partly through lower subsequent anxiety, while social support may shape the PA-anxiety link. Because anxiety and NSSI were measured concurrently at follow-up, the indirect effect should not be interpreted as definitive causal mediation. Multi-wave and intervention studies are needed to establish temporal ordering and causality.

## 1 Introduction

Non-suicidal self-injury (NSSI) refers to deliberate, direct damage to body tissue without suicidal intent and outside socially sanctioned practices (1, 38). Although NSSI is conceptually distinct from suicidal behavior, it is clinically important in its own right and is associated with later suicidal thoughts and attempts (2, 3). Reviews and epidemiological syntheses indicate that NSSI commonly emerges during adolescence, with prevalence estimates varying substantially across definitions, assessment methods, and sampling frames (4–7). In China, a meta-analysis of middle- and high-school samples estimated a pooled prevalence of 22.37%, while also documenting considerable between-study heterogeneity (8). These findings underscore the importance of identifying modifiable factors that may be linked to lower NSSI risk during a developmental period characterized by rapid biological, emotional, and social change.

NSSI is heterogeneous in function and course. Functional accounts emphasize that self-injury can provide short-term relief from aversive affect, interrupt distressing cognitive states, or serve interpersonal functions, even though it carries substantial longer-term costs (1, 9, 10). Emotional disorders are overrepresented among individuals who engage in NSSI, and meta-analytic evidence indicates meaningful associations between NSSI and anxiety- and mood-related disorders (11). Longitudinal research further shows that previous NSSI and broader psychopathology are important predictors of subsequent self-injury (12, 13), whereas meta-analytic work highlights the multifactorial nature of risk (14). Accordingly, prevention research increasingly focuses on modifiable behavioral and psychosocial correlates rather than any single putative cause.

Physical activity (PA) is one candidate behavioral factor. International guidelines recommend regular moderate-to-vigorous PA for children and adolescents because of broad physical and mental health benefits (15). Reviews of youth research suggest that PA is associated with better mental health, although the strength of evidence differs across outcomes and study designs and causal conclusions remain constrained by the predominance of observational studies (16–18). Potential mechanisms include changes in stress-related physiology, affect regulation, self-perceptions, mastery, and social connection (19). Sport participation may also provide structured opportunities for peer affiliation and belonging, which can be psychologically beneficial when the social environment is supportive (20).

A small but growing literature has examined PA in relation to NSSI more directly. Boone and Brausch (21) found that greater PA was associated with lower NSSI frequency in a mixed high-school and undergraduate sample. More recent studies in Chinese adolescents have reported inverse associations between physical exercise and NSSI and have proposed psychosocial mechanisms involving perceived social support, self-concept, psychological capital, and relative deprivation (22, 23). These studies are informative, but much of the evidence remains cross-sectional. The temporal direction of association is therefore uncertain: lower PA may precede later distress and NSSI, but psychological difficulties associated with NSSI may also reduce motivation or opportunity to be active.

Anxiety symptoms are a theoretically plausible emotional pathway linking PA to NSSI. Experiential avoidance and functional models propose that NSSI can be negatively reinforced when it temporarily reduces intense negative affect (9, 10). Anxiety is also strongly related to NSSI across emotional-disorder research (11). At the same time, youth PA is associated with lower anxiety and broader improvements in emotional well-being, although effect estimates vary across studies and intervention contexts (16, 18). A prospective pattern in which higher PA is followed by lower anxiety, which in turn is associated with lower NSSI, would therefore be consistent with established theories of affect regulation. Importantly, however, a two-wave design cannot establish full temporal mediation when the mediator and outcome are measured at the same follow-up assessment (24). The present study consequently treats the indirect effect as a prospective association consistent with mediation rather than as evidence of a causal mechanism.

The social context may further condition the association between PA and anxiety. The stress-buffering hypothesis proposes that perceived or available support can reduce the psychological impact of stress, particularly when support resources match the demands faced by the individual (25). Meta-analytic evidence links social support with well-being in children and adolescents and with lower depressive symptoms across youth samples (26, 27). Within PA settings, supportive peers, teachers, and family members may reinforce participation, increase perceived competence and belonging, and make activity experiences more emotionally restorative (20). Thus, adolescents with stronger social support may derive greater anxiety-related benefit from PA than those whose activity occurs in less supportive contexts.

The present study used a two-wave, six-month design to examine these questions in a school-based Chinese sample. Baseline levels of the relevant outcomes were included as autoregressive covariates to reduce, though not eliminate, ambiguity about temporal ordering (24). Four hypotheses were evaluated: H1, higher T1 PA would be associated with lower T2 NSSI after adjustment for T1 NSSI; H2, T2 anxiety would carry a significant indirect association between T1 PA and T2 NSSI after adjustment for T1 anxiety and T1 NSSI; H3, T1 social support would moderate the T1 PA-T2 anxiety association, with a stronger inverse association at higher support; and H4, T1 social support would moderate the T2 anxiety-T2 NSSI association.

## 2 Method

### 2.1 Participants and sampling

The study used a multistage school-based cluster sampling strategy with geographic and educational stratification. Participants were recruited from educational settings in Anhui and Jiangxi Provinces. Urban and rural districts were represented, and schools were sampled across junior secondary, senior secondary, and tertiary educational levels. Within participating schools, intact classes were selected as the final sampling units. This design was intended to increase demographic and educational heterogeneity relative to a single-site convenience sample; it should not be interpreted as a nationally representative probability sample of Chinese adolescents.

At T1 (October 2022), 896 students completed questionnaires assessing demographic characteristics, PA, social support, anxiety symptoms, and NSSI. Six months later, at T2 (April 2023), the cohort was invited to complete follow-up measures of anxiety and NSSI. Anonymous records were linked across waves using self-generated identification codes. The original data-cleaning protocol excluded unmatched records, cases with more than 10% missing questionnaire data, and responses showing evident straight-lining. The final analytic sample comprised 756 participants, corresponding to a retention rate of 84.4%. Participants in the retained sample had a mean age of 17.36 years (SD = 2.58), 391 (51.7%) were female, and 500 (66.1%) lived in urban areas.

### 2.2 Measures

#### 2.2.1 Physical activity at T1

PA during the previous 7 days was assessed with the Chinese short form of the International Physical Activity Questionnaire (IPAQ-C). The short IPAQ has been evaluated internationally (28), and the Chinese version has demonstrated acceptable test–retest reliability for total PA, although agreement with accelerometry is limited and subcomponent validity is weaker (29). Participants reported the frequency and duration of vigorous activity, moderate activity, and walking. In accordance with standard IPAQ scoring, weekly metabolic equivalent (MET)-minutes were calculated using weights of 8.0, 4.0, and 3.3 METs for vigorous activity, moderate activity, and walking, respectively. Because the IPAQ-SF is a behavioral index composed of activity domains rather than a reflective psychometric scale, internal-consistency coefficients were not used to characterize its reliability.

#### 2.2.2 Non-suicidal self-injury at T1 and T2

NSSI was assessed at both waves with the behavioral section of the Adolescent Non-suicidal Self-injury Assessment Questionnaire (ANSAQ), developed for Chinese adolescents (30). The instrument assesses 12 forms of self-injurious behavior, including behaviors involving relatively less visible tissue damage (e.g., severe scratching) and more overt tissue damage (e.g., cutting or burning). Frequency responses were recorded on a five-point scale from 0 (no occurrence) to 4 (more than 20 times), and a composite score was used in the analyses, with higher values indicating more frequent or severe NSSI. Internal consistency in the present sample was high at T1 (Cronbach’s alpha = 0.925) and T2 (alpha = 0.931).

#### 2.2.3 Anxiety symptoms at T1 and T2

Anxiety symptoms were measured at both waves with the seven-item Generalized Anxiety Disorder scale (GAD-7; (31)), which has shown good reliability and validity in Chinese clinical samples (32). Items assess the frequency of core anxiety symptoms over the previous 2 weeks. The standard GAD-7 uses 0–3 response coding; in the study dataset, responses were stored using an equivalent 1–4 coding, producing totals from 7 to 28. This additive shift changes the intercept but not correlations, standardized regression coefficients, or the interpretation of between-person differences. Internal consistency was high at T1 (alpha = 0.917) and T2 (alpha = 0.920).

#### 2.2.4 Social support at T1

Perceived social support was assessed with a 17-item Chinese student social support scale based on the three-domain framework of subjective support, objective support, and support utilization developed by Ye and Dai (33). Items were rated from 1 (does not fit) to 5 (fits very well), with higher total scores indicating greater perceived and utilized support. The original scale was validated in university students and has subsequently been applied in younger student samples; given the broad age range of the present sample, this measurement history is considered when interpreting generalizability. Internal consistency for the total score in the present sample was alpha = 0.920.

### 2.3 Procedure and ethics

Trained research assistants administered the questionnaires in classroom groups using standardized instructions. Participants were informed that participation was voluntary, that they could discontinue without penalty, and that questionnaire data would be treated confidentially. Written informed consent was obtained from participants; for participants younger than 18 years, written parental or guardian consent was also obtained. The study was conducted in accordance with the Declaration of Helsinki and was approved by the Ethics Committee of China University of Mining and Technology (CUMT-IRB-2022-007).

### 2.4 Data analysis

Analyses were conducted in SPSS 26.0 with PROCESS 4.0. Retained and attrited participants were compared on baseline demographic and study variables using independent-samples *t* tests for continuous variables and chi-square tests for categorical variables. Missingness in the retained analytic dataset was below 2%. After Little’s (34) test did not provide evidence against a missing-completely-at-random pattern, missing values were handled using expectation–maximization procedures.

Descriptive statistics and partial correlations were calculated for the core variables, adjusting correlations for age, gender, and urban/rural residence. To examine prospective associations, regression equations included baseline levels of the relevant outcome wherever possible. Specifically, T1 anxiety was entered when predicting T2 anxiety, and T1 NSSI was entered when predicting T2 NSSI. This autoregressive adjustment estimates associations with residualized follow-up differences conditional on baseline status, but it does not by itself establish causal change (24).

The indirect association of T1 PA with T2 NSSI through T2 anxiety was evaluated with regression-based conditional process procedures and 5,000 bootstrap resamples (35). Age, gender, urban/rural residence, T1 anxiety, and T1 NSSI were included as covariates in the relevant equations. Continuous predictors were mean-centered before interaction terms were formed. First-stage moderation was evaluated with the T1 PA × T1 social support interaction predicting T2 anxiety; second-stage moderation was evaluated with the T2 anxiety × T1 social support interaction predicting T2 NSSI. Because the design included only two waves and anxiety and NSSI were assessed concurrently at T2, the analysis is described as a half-longitudinal or prospective indirect-effect model rather than a definitive longitudinal mediation test (24).

As an ancillary diagnostic, a single-factor exploratory test was used to examine whether one factor dominated the covariance among self-report measures; the first factor accounted for 27.82% of the variance. This diagnostic is included only as an ancillary check and is not treated as evidence that common-method variance is absent, because single-factor tests have limited sensitivity (36). Two-sided *p* values are reported, and 95% confidence intervals (CIs) are used for bootstrap effects.

## 3 Results

### 3.1 Attrition analysis

Baseline comparisons between the 756 retained participants and 140 participants not retained at T2 are shown in Table 1. None of the reported comparisons reached statistical significance (all *p* > 0.05). Thus, the observed baseline variables did not provide evidence of systematic attrition; nevertheless, the possibility of attrition related to unmeasured characteristics cannot be excluded.

**Table 1:** Demographic characteristics and attrition comparisons.

Variable	Retained (*N* = 756)	Attrited (*N* = 140)	Chi-square or *t*	*p*
Gender, *n* (%)			0.84	0.358
Male	365 (48.3%)	73 (52.1%)		
Female	391 (51.7%)	67 (47.9%)		
Location, *n* (%)			1.12	0.289
Urban	500 (66.1%)	87 (62.1%)		
Rural	256 (33.9%)	53 (37.9%)		
Age, years, M (SD)	17.36 (2.58)	17.51 (2.49)	−0.62	0.536
T1 PA, M (SD)	2245.3 (1082.1)	2150.8 (1110.4)	1.05	0.294
T1 anxiety, M (SD)	11.82 (4.88)	12.20 (5.01)	−0.88	0.380
T1 NSSI, M (SD)	0.91 (1.25)	1.04 (1.31)	−1.12	0.263

PA, physical activity; NSSI, non-suicidal self-injury. Chi-square statistics are reported for categorical variables and *t* statistics for continuous variables.

### 3.2 Descriptive statistics and partial correlations

Table 2 presents descriptive statistics and partial correlations adjusted for age, gender, and urban/rural residence. T1 PA was inversely correlated with T2 anxiety (*r* = −0.35, *p* < 0.001) and T2 NSSI (*r* = −0.37, *p* < 0.001). T1 social support was also inversely correlated with anxiety and NSSI at both waves. Anxiety and NSSI showed substantial rank-order stability across the six-month interval (*r* = 0.62 and *r* = 0.65, respectively, both *p* < 0.001).

**Table 2:** Descriptive statistics and partial correlations among core variables.

Variable	M (SD)	1	2	3	4	5	6
1. T1 PA	2245.3 (1082.1)	1.00					
2. T1 social support	58.74 (10.81)	0.28^***^	1.00				
3. T1 anxiety	11.82 (4.88)	−0.30^***^	−0.29^***^	1.00			
4. T1 NSSI	0.91 (1.25)	−0.34^***^	−0.25^***^	0.43^***^	1.00		
5. T2 anxiety	11.55 (4.90)	−0.35^***^	−0.31^***^	0.62^***^	0.40^***^	1.00	
6. T2 NSSI	0.88 (1.22)	−0.37^***^	−0.28^***^	0.39^***^	0.65^***^	0.46^***^	1.00

*N* = 756. Partial correlations control for age, gender, and urban/rural residence. PA, physical activity; NSSI, non-suicidal self-injury. ^***^p < 0.001.

### 3.3 Autoregressive regression models and moderation tests

Hierarchical regression results are summarized in Table 3. After adjustment for T1 anxiety and demographic covariates, higher T1 PA was associated with lower T2 anxiety (*β* = −0.21, *p* < 0.001), and higher T1 social support was independently associated with lower T2 anxiety (*β* = −0.15, *p* < 0.001). The T1 PA × T1 social support interaction added 1.5% explained variance and was statistically significant (*β* = −0.14, *p* = 0.001), supporting the presence of first-stage moderation. The direction of the interaction is interpreted below using the reported simple slopes.

**Table 3:** Hierarchical autoregressive regression models for anxiety and NSSI at T2.

Outcome	Step/predictor	*β*	*t*	*p*	*R* ^2^	Δ*R*^2^
T2 anxiety	Step 1: autoregressive + controls				0.392	0.392^***^
	T1 anxiety	0.58	19.82	<0.001		
	Age, gender, location	–	–	>0.05		
	Step 2: main effects				0.435	0.043^***^
	T1 PA	−0.21	−6.54	<0.001		
	T1 social support	−0.15	−4.89	<0.001		
	Step 3: interaction				0.450	0.015^**^
	T1 PA × T1 social support	0.14	3.96	0.003		
T2 NSSI	Step 1: autoregressive + controls				0.428	0.428^***^
	T1 NSSI	0.61	21.05	<0.001		
	Age, gender, location	–	–	>0.05		
	Step 2: main effects				0.495	0.067^***^
	T1 PA	*β* = −0.12	*t* = −3.78	<0.001		
	T2 anxiety	0.28	8.44	<0.001		
	T1 social support	−0.06	−1.91	0.057		
	Step 3: interaction				0.497	0.002
	T2 anxiety × T1 social support	−0.04	−1.08	0.281		

*N* = 756. Standardized regression coefficients are reported. Continuous predictors were mean-centered before interaction terms were formed. PA, physical activity; NSSI, non-suicidal self-injury. ^**^p < 0.01, ^***^p < 0.001.

When predicting T2 NSSI, T1 NSSI showed strong temporal stability (*β* = 0.61, *p* < 0.001). T1 PA remained inversely associated with T2 NSSI (*β* = −0.12, *p* = 0.002), whereas T2 anxiety was positively associated with T2 NSSI (*β* = 0.28, *p* < 0.001). T1 social support did not show an independent association at the conventional 0.05 level in this equation (*β* = −0.06, *p* = 0.057). The T2 anxiety × T1 social support interaction was not significant (*β* = −0.04, *p* = 0.281); H4 was therefore not supported.

### 3.4 Prospective indirect association through anxiety

Bootstrap estimates are shown in Table 4. The reported total association of T1 PA with T2 NSSI was −0.25 [95% CI (−0.34, −0.16)]. After T2 anxiety was included, the direct association remained negative [−0.13, 95% CI (−0.20, −0.06)]. The indirect association through T2 anxiety was −0.12 [bootstrap SE = 0.03, 95% CI (−0.18, −0.06)]. Because the bootstrap CI excluded zero, the data were consistent with an indirect pathway in which greater T1 PA was associated with lower T2 anxiety, which in turn was associated with lower T2 NSSI. The indirect component represented 48.0% of the reported total association. Given the concurrent measurement of anxiety and NSSI at T2, this proportion should be viewed as descriptive rather than as a causal decomposition.

**Table 4:** Bootstrap estimates for the prospective indirect association through T2 anxiety.

Effect	Path	Estimate	Boot SE	95% CI	% total
Total	T1 PA → T2 NSSI	−0.25	0.04	[−0.34, −0.16]	–
Direct	T1 PA → T2 NSSI	−0.13	0.03	[−0.20, −0.06]	52.0%
Indirect	T1 PA → T2 anxiety → T2 NSSI	−0.12	0.03	[−0.18, −0.06]	48.0%

*N* = 756. Models controlled for T1 NSSI, T1 anxiety, age, gender, and urban/rural residence as appropriate. Bootstrap estimates are based on 5,000 resamples. PA, physical activity; NSSI, non-suicidal self-injury; CI, confidence interval.

Figure 1 summarizes the principal coefficients in the conditional process model, including the direct PA-NSSI association, the PA-anxiety and anxiety-NSSI paths, and the first-stage PA × social support interaction.

**Figure 1:**
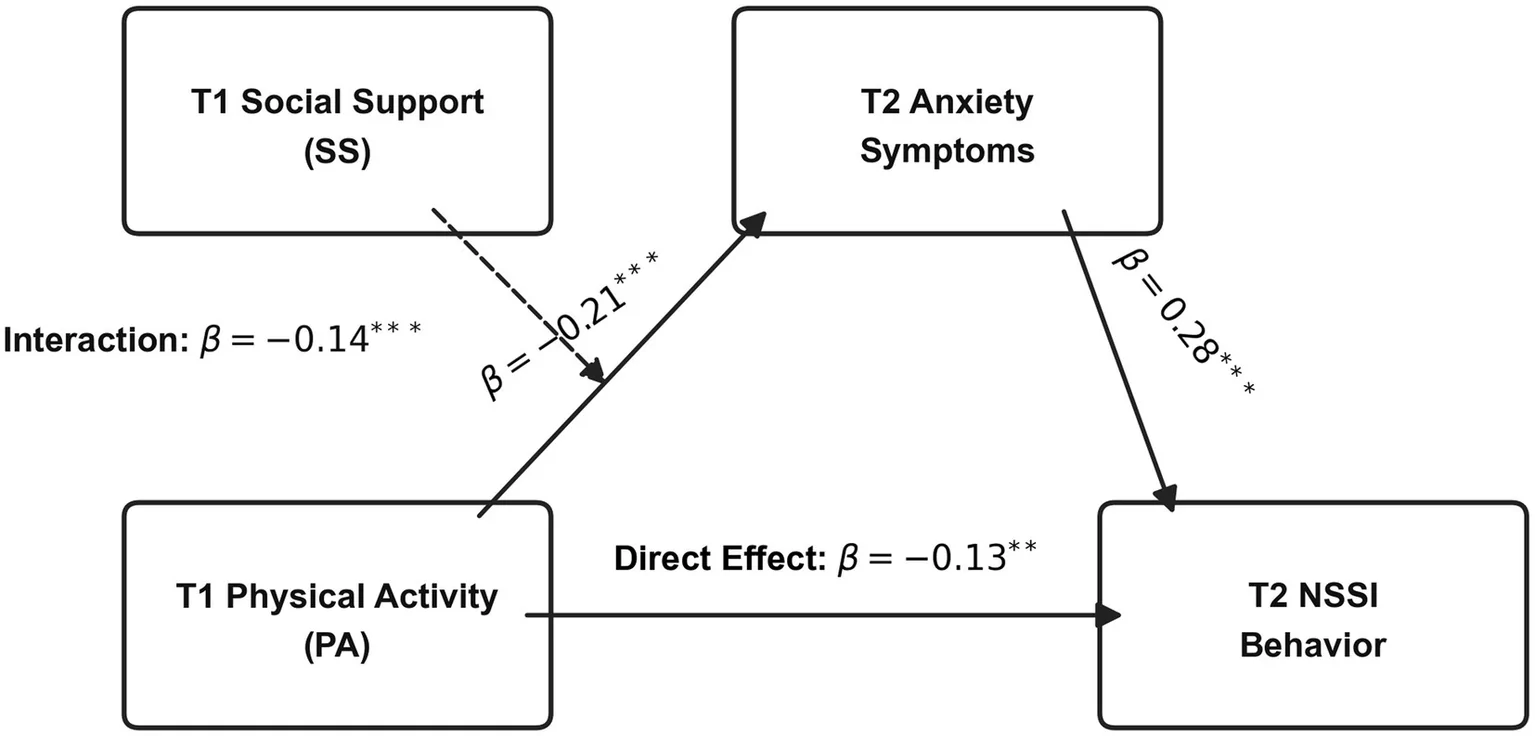
Estimated conditional process model linking T1 physical activity with T2 NSSI through T2 anxiety symptoms. Coefficients are standardized regression estimates from the present analysis. The model controls for T1 NSSI, T1 anxiety, age, gender, and location. PA, physical activity; SS, social support; NSSI, non-suicidal self-injury. ^**^p < 0.01, ^***^p < 0.001.

### 3.5 Simple-slope analysis of the PA × social support interaction

The significant first-stage interaction was probed at one standard deviation above and below the T1 social support mean. At high social support (+1 SD), T1 PA was inversely associated with T2 anxiety [*β* = −0.32, *t* = −7.85, *p* < 0.001, 95% CI (−0.41, −0.23)]. At low social support (−1 SD), the association remained significant but was smaller in magnitude [*β* = −0.15, *t* = −3.42, *p* = 0.001, 95% CI (−0.24, −0.06)]. Thus, the simple-slope pattern was consistent with H3: higher social support was associated with a steeper inverse relation between PA and subsequent anxiety. Figure 2 displays the model-implied pattern across low and high levels of PA.

**Figure 2:**
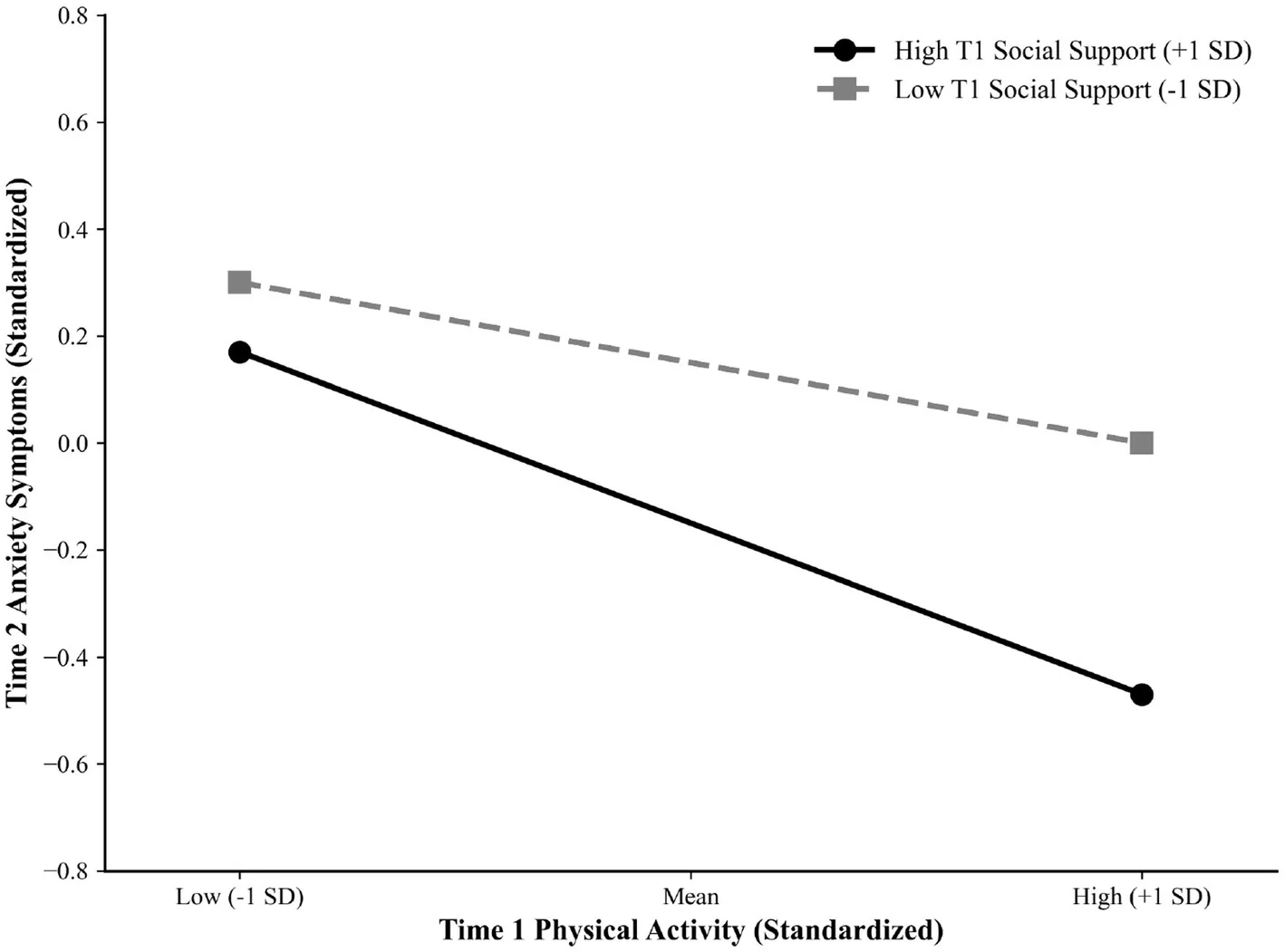
Simple slopes of T1 physical activity predicting T2 anxiety at low and high T1 social support. Lines depict model-implied standardized T2 anxiety values at low (−1 SD) and high (+1 SD) T1 social support across low (−1 SD), mean, and high (+1 SD) T1 physical activity.

## 4 Discussion

This two-wave study examined whether PA was prospectively associated with NSSI and whether anxiety symptoms and social support helped characterize that association. Three findings were central. First, higher T1 PA was associated with lower T2 NSSI after adjustment for baseline NSSI and demographic covariates. Second, a significant indirect association through T2 anxiety was observed, with anxiety accounting descriptively for 48% of the reported total association. Third, social support moderated the PA-anxiety association, whereas there was no evidence that it moderated the anxiety-NSSI association. Together, the results are compatible with a model in which PA and social resources operate partly upstream of later emotional distress. At the same time, the observational design and the concurrent T2 measurement of anxiety and NSSI require cautious interpretation.

### 4.1 Physical activity and subsequent NSSI

The inverse prospective association between T1 PA and T2 NSSI extends a literature that has largely relied on cross-sectional designs. Prior work has linked greater PA with lower NSSI frequency (21), and recent Chinese studies similarly report inverse PA-NSSI associations while identifying psychosocial correlates that may help explain them (22, 23). The present study adds temporal separation between baseline PA and follow-up NSSI and adjusts for baseline NSSI, which reduces the likelihood that the association is explained solely by pre-existing differences in self-injury.

Several mechanisms are plausible, but the data do not isolate them. Reviews of youth PA identify psychosocial processes such as self-efficacy, self-perceptions, social connection, and affect regulation, together with possible neurobiological and stress-related mechanisms (17–19). PA may also provide routine, behavioral activation, and opportunities for rewarding social engagement. Importantly, these mechanisms should not be treated as established explanations of the present PA-NSSI coefficient. Experimental or intensive longitudinal designs are needed to determine whether changing PA produces changes in NSSI and through which processes.

### 4.2 Anxiety as a candidate indirect pathway

The indirect-effect results were consistent with anxiety as one candidate emotional pathway. This interpretation aligns with functional and experiential-avoidance accounts of NSSI, in which self-injury can be reinforced because it temporarily changes intolerable affective states (1, 9, 10). It is also consistent with meta-analytic evidence linking NSSI with emotional disorders, including anxiety-related conditions (11). In the present data, higher T1 PA was associated with lower T2 anxiety even after T1 anxiety was controlled, and higher T2 anxiety was associated with greater T2 NSSI after baseline NSSI was controlled.

The PA-anxiety link is plausible in light of broader youth mental health evidence. Reviews suggest that PA can be associated with lower anxiety and better emotional well-being, but the magnitude and certainty of effects vary across measurement approaches, populations, and interventions (16–18). Mechanistic reviews likewise emphasize that no single biological or psychosocial route is sufficient to explain all mental health effects of PA (19). Accordingly, anxiety should be viewed as one potential pathway among several rather than the unique mechanism linking PA and NSSI.

A further qualification is essential. Although the model controls T1 anxiety and T1 NSSI, anxiety and NSSI were both measured at T2. Consequently, the design does not establish that change in anxiety occurred before change in NSSI. Cole and Maxwell (24) note that stronger longitudinal mediation tests require temporal separation of the predictor, mediator, and outcome, ideally across three or more waves. The current indirect association is therefore informative about covariance in prospective change but cannot confirm the causal sequence implied by mediation.

### 4.3 Social support as a boundary condition

Social support moderated the prospective association between PA and anxiety. The reported simple slopes indicated that the inverse PA-anxiety association was stronger among participants with higher baseline social support. This pattern is broadly consistent with the stress-buffering hypothesis (25) and with meta-analytic evidence that perceived support is linked to better youth well-being (26, 27). It is also compatible with sport research showing that participation can confer social and psychological benefits when activity contexts foster belonging and positive relationships (20). PA may therefore be more emotionally beneficial when it is embedded in relationships that provide encouragement, companionship, and a sense of safety.

By contrast, the T2 anxiety × T1 social support interaction did not significantly predict T2 NSSI. This null result should not be interpreted as evidence that social support is irrelevant once anxiety is present. Rather, the present measure of general social support did not detect a reliable difference in the anxiety-NSSI association after the included covariates were controlled. Support may matter through other pathways, may operate differently for family, peer, and school sources, or may depend on whether adolescents disclose distress and are able to mobilize available support. The absence of second-stage moderation therefore narrows the evidence for the proposed model but does not imply a general threshold at which social resources cease to matter.

### 4.4 Implications for prevention and practice

The results support a cautious prevention-oriented interpretation. PA is low cost at the population level, can be embedded in school routines, and is already recommended for adolescent health (15). The moderation pattern suggests that programs may be more useful when they attend not only to activity dose but also to the quality of the social setting in which activity occurs. Team-based or group activities, supportive physical education climates, and family encouragement may be especially relevant when the goal is to promote emotional well-being rather than simply increase energy expenditure.

At the same time, PA should not be positioned as a stand-alone treatment for adolescents who are already engaging in NSSI. Clinical guidance emphasizes direct assessment of NSSI and suicidality, attention to comorbid psychopathology, and psychotherapeutic approaches that target emotion regulation and related difficulties (37). Because NSSI is a marker of elevated future suicide risk (2, 3), school or community PA initiatives should complement, not substitute for, appropriate mental health evaluation and care.

### 4.5 Strengths, limitations, and future directions

Several strengths merit note. The six-month follow-up and adjustment for baseline anxiety and NSSI improve on purely cross-sectional comparisons, while the multistage school-based sampling strategy captured participants from multiple educational levels and urban and rural settings. The analyses also distinguished first- and second-stage moderation instead of assuming that social support operates uniformly across the model.

The limitations are equally important. First, two waves are insufficient for a strong temporal test of mediation because the proposed mediator and outcome were measured concurrently at T2. Three-wave or denser longitudinal designs should measure PA, anxiety, and NSSI at sequential occasions and test reciprocal paths. Second, all core variables were self-reported. Shared method variance, recall error, and social desirability may influence observed associations; the single-factor diagnostic cannot rule these problems out (36). Future work should combine self-report with accelerometry or wearable activity measures and ecological momentary assessment of affect and NSSI urges.

Third, the sampling frame was limited to educational settings in two Chinese provinces. Although the design was more heterogeneous than a single-site convenience sample, it does not justify national representativeness. Fourth, participants were clustered within classes and schools, whereas the reported PROCESS regressions were conducted at the individual level. If intraclass correlations were nontrivial, conventional standard errors may be optimistic; future analyses should use multilevel models or cluster-robust standard errors. Fifth, the social support instrument was originally validated in university students (33) while the present sample spanned a broader educational age range. Replication should evaluate measurement invariance and, ideally, distinguish family, peer, and teacher support.

Finally, NSSI scores are often zero-inflated and positively skewed. The reported analyses used linear regression on the study composite, which facilitates comparison of standardized paths but may not optimally represent the distribution of NSSI. Future studies should examine whether results replicate using models suited to counts, ordinal outcomes, or zero-inflated distributions and should preregister analytic decisions.

## 5 Conclusion

In this two-wave Chinese cohort, higher baseline PA was associated with lower NSSI 6 months later after baseline NSSI was taken into account. A significant prospective indirect association through anxiety was also observed, and social support moderated the PA-anxiety association but not the anxiety-NSSI association. These findings suggest that PA, anxiety, and social support may be meaningfully linked within adolescent self-injury risk processes. They do not establish that PA causally prevents NSSI or that anxiety is a temporally confirmed mediator. Stronger multi-wave and intervention research is required before such causal claims are warranted. From a prevention perspective, the results nevertheless support further evaluation of PA programs that are embedded in supportive social environments and integrated with evidence-based mental health care for adolescents at elevated NSSI risk.

## Data Availability

The datasets presented in this article are not publicly available because they contain sensitive participant information and are subject to privacy restrictions. Requests to access the datasets should be directed to Jianyi Li, lijianyikf@foxmail.com. De-identified data may be made available upon reasonable request, subject to applicable privacy and ethical restrictions.
